# COVID-19 related neurological manifestations in Parkinson’s disease: has ferroptosis been a suspect?

**DOI:** 10.1038/s41420-024-01915-6

**Published:** 2024-03-19

**Authors:** Fengju Jia, Jing Han

**Affiliations:** School of Nursing, Qingdao University, No. 308 Ningxia Road, Qingdao, 266071 China

**Keywords:** Parkinson's disease, Viral infection

## Abstract

A rising number of patient cases point to a probable link between SARS-CoV-2 infection and Parkinson’s disease (PD), yet the mechanisms by which SARS-CoV-2 affects the brain and generates neuropsychiatric symptoms in COVID-19 patients remain unknown. Ferroptosis, a distinct iron-dependent non-apoptotic type of cell death characterized by lipid peroxidation and glutathione depletion, a key factor in neurological disorders. Ferroptosis may have a pathogenic role in COVID-19, according to recent findings, however its potential contributions to COVID-19-related PD have not yet been investigated. This review covers potential paths for SARS-CoV-2 infection of the brain. Among these putative processes, ferroptosis may contribute to the etiology of COVID-19-associated PD, potentially providing therapeutic methods.

## Facts

- Ferroptosis may have a pathogenic role in COVID-19.
- The brain is infected by SARS-CoV-2 via potential paths.
- Ferroptosis may contribute to the etiology of COVID-19-associated Parkinson’s disease.

## Open questions

- What are the links between COVID-19 and Parkinson’s disease?
- What are the potential role of ferroptosis in COVID-19-related Parkinson’s disease?

## Introduction

Global anxiety and an economic catastrophe have been brought on by the severe acute respiratory syndrome coronavirus 2 (SARS-CoV-2) epidemic, often known as the 2019 new coronavirus disease (COVID-19) pandemic [1]. As of 17 December 2023, there have been 772 million confirmed cases of COVID-19 worldwide, including 6.9 million deaths, reported to the World Health Organization [2]. The impact of COVID-19 has been unsurpassed thus far, and its long-term effects might be far more disastrous [3, 4]. The SARS-CoV-2 virus, which causes the current COVID-19 pandemic, affects more than just the respiratory system, it also affects other organs and tissues [5]. SARS-CoV-2 has recently been discovered in neurons in several parts of the brain, including substantia nigra [6, 7]. Several individuals with SARS-CoV-2 infections have reported experiencing acute and subacute neurological complications [8–10]. Due to a number of causes that cause a reduction in the dopaminergic neurons of the substantia nigra followed by striatal dopamine depletion, patients with Parkinson’s disease (PD) have a variety of motor and non-motor impairments [11]. In this review, we explore current evidence indicating a potential pathogenic links between COVID-19 and PD, and provide directions for potential therapeutic approaches that target ferroptosis.

## COVID-19 and PD: a more defined picture

While the specific mechanism causing the presumable degradation of nigrostriatal dopaminergic neurons after a viral infection is still unknown, viral infection is receiving more and more attention as a cause of PD [12, 13]. Studies have indicated that SARS-CoV-2 can infiltrate the central nervous system (CNS) and cause additional neurological dysfunction in a considerable percentage of infected patients [9, 14].

36% of SARS-CoV-2 infections experience neurological symptoms during the acute stage, 25% of which may be linked to CNS involvement directly [15]. The substantia nigra are particularly susceptible to SARS-CoV-2 [16]. Not all neuronal populations are equally prone to degeneration. Due to their intrinsic characteristics, such as high energy needs to support heightened basal oxidative phosphorylation in the mitochondria, high axon terminal density, and substantial axonal arborization, dopaminergic neurons are particularly susceptible to degeneration. Interestingly, at least 20 cases show that COVID-19 patients experienced clinical parkinsonism following SARS-CoV-2 infection [17], pointing to a potential link between COVID-19 infection and newly formed parkinsonism.

## COVID-19 neurotropism and PD: exploring the links

The CNS has been disrupted by COVID-19 in a variety of ways, including direct SARS-CoV-2 invasion of neuronal cells, huge inflammatory elements driven by severe systemic inflammation flowing into brains, respiratory failure linked brain ischemia, etc [18–20].

### Olfactory bulb

Up to 20% of adults with COVID-19 infected individuals exhibit anosmia/hyposmia and ageusia, which is a neurological symptom, at an early stage of the viral illness [21]. Anosmia, however, is a well-known precursor signal for PD development [22]. Furthermore, studies showed that SARS-CoV-2 is able to enter directly through the olfactory neurons and, intriguingly, without first affecting the lungs [23]. Impaired neurogenesis in the olfactory system may bing on the anosmia in COVID-19 and PD [24]. SARS-CoV-2 may have direct access to brain areas for the development of PD, according to neuropathological investigations utilizing immunostaining of α-synuclein aggregates that imply that PD starts in either the olfactory or intestinal neurons and progresses to the brain. Apparently, SARS-CoV-2 may enter the brain by the olfactory pathways and spread to the basal ganglia, brainstem, and piriform and infralimbic cortex [25].

### Gut microbiome and gut physiology

A systemic inflammatory state that SARS-CoV-2 induces may enhance the risk for PD in addition to the direct invasion of the CNS [26]. Gastrointestinal symptoms are also brought on by COVID-19, and SARS-CoV-2 RNA has been found in the feces of infected individuals, suggesting that the virus is intestinal in origin. According to a recent study [25], enterocytes are the main target cells of SARS-CoV-2 and they respond to the infection by triggering a powerful inflammatory response. These results could emphasize COVID-19’s possible function as a PD risk factor even more [27].

Another idea contends that the gut microbiota is the starting point of the inflammatory process that results in PD [28]. Surprisingly, the neurological symptoms and gut microbiota changes seen in patients of COVID-19 are also frequently present in patients of PD [29]. Moreover, SARS-CoV-2 intestinal infection may change gut physiology in general and gut microbiota [30], impacting all aspects that “peripherally” contribute to the etiology and development of PD [31].

### Angiotensinconverting enzyme 2 (ACE2)

One of the major receptors that facilitates the entry of SARS-CoV-2 into human cells, is the ACE2 receptor [32, 33]. After infection, COVID-19 has a greater affinity for protein S, which allows the viral glycoprotein to attach to host cells ACE2 [34–36]. These receptors are widely distributed on neurons and glial cells of many brain areas, including cerebral cortex, striatum, substantia nigra, and brain stem [37]. In dopaminergic neurons, which are diminished in PD patients, ACE2 is significantly expressed and may contribute to the aggravation of pre-existing symptoms or a more severe COVID-19 infection [38]. Due to ACE2 and DOPA decarboxylase co-express and co-regulate in non-neuronal cell types, the dopamine synthesis route may be implicated in the pathogenesis of COVID-19 [39, 40]. ACE2 expression is downregulated by SARS-CoV infection, which may contribute to the impairment of dopamine production [41–43]. There is evidence that the expression levels of ACE2 in brains of PD patients have reduced, causing dopaminergic neuron loss and degeneration [44–46].

### COVID-19 and PD: shared inflammatory pathways under oxidative stress

SARS-CoV-2 has the capacity to generate a dysregulation of cytokines-“cytokine storm“ [47]. In order to regulate the infection that might damage neurons, cytokines such interleukin receptor-2, interleukin-6, and tumor necrosis factor are released by infected neurons [48]. The development of both COVID-19 and PD may be significantly influenced by oxidative stress and cytokine storm. Moreover, the blood-brain barrier (BBB) may break down as a result of the severe systemic inflammatory response brought on by viral infection. As a result, peripheral cytokines may be able to enter the CNS, where they may cause or exacerbate neuroinflammation [49]. Virus-induced inflammation is thought to contribute to neurodegeneration [50], as is “multiple hit” damage [51]. Just like the “two hit” concept of PD, the COVID-19 infection might have served as an infectious second hit [52]. The inflammatory response induced by acute or chronic infection may initiate or accelerate early and subclinical processes underlying the early stages of PD. Additionally, research on neurodegenerative diseases and other viral infections indicates that systemic inflammation brought on by SARS-CoV-2 infection may further contribute to neuroinflammatory processes and increase susceptibility to PD [53]. Also, the discovery of possible therapeutic strategies for the treatment of COVID-19 and PD is aided by the targeted suppression of caspases and nuclear factor kappa B activation [54]. Due to the anti-inflammatory properties of vitamin D3, regular supplementation with 2000–5000 IU/day of D3 may help older persons with PD reduce the evolution of their condition and may also provide further protection against COVID-19 [55].

### α-synuclein

The nigrostriatal dopaminergic system suffers from neurodegeneration brought on by α-synuclein, which is clinically evident as the usual PD/parkinsonian symptoms. α-synuclein overexpression is thought to be related to SARS-CoV-2 infection. SARS-CoV-2 neuroinfection causes increased levels of α-synuclein [46, 47]. Indeed, SARS-CoV-2 infection seems to cause α-synuclein aggregation in the brains of COVID-19 cases [56]. In vitro experiments have demonstrated that the SARS-CoV-2 N-protein speeds up the aggregation of α-synuclein [57]. N-protein microinjection disrupted the α-synuclein proteostasis and enhanced cell mortality in SH-SY5Y cells [57]. Besides. the SARS-CoV-2 infection may potentially hinder the removal of α-synuclein. The overexpression of α-synuclein, which may play a role in the immune response [58], may then cause microglia to become active [59]. Microglia cells would amp up the inflammatory response and release inflammatory cytokines and chemokines, which would result in neuronal death [60, 61]. Furthermore, glutamate excitotoxicity, which is connected to neuronal degeneration, may result from neuroimmune reactions to an infection [62, 63]. Therefore, SARS-CoV-2 infection seems to affect α-synuclein and death of dopaminergic neurons, which is known to cause PD.

### Glial cells

Astrocytes and microglia, in particular, are now considered to play a significant role in both beneficial and negative host responses during CNS illness states [64]. With the increasing number of individuals infected and re-infected across the world, microglia may have a role in the pathophysiology of post-COVID-19 neurological diseases, including PD [65]. By up-regulation of inflammatory cytokine genes and enhanced BBB permeability, reactive astrocytes are frequently engaged in processes of neurodegeneration and neuroinflammation. When pathogen-derived or endogenous ligands are detected by injured cells, pattern recognition receptors (PRRs), which are produced by astrocytes and microglia, start the innate immune response [66]. Toll-like receptors (TLRs), a well-known class of PRRs, might be involved in the cytokine storm brought on by SARS-CoV-2 [67]. In fact, TLR4, are likely to detect SARS-CoV-2-derived molecular patterns and trigger an inflammatory response. TLR2 and TLR7/TLR8 are also activated by SARS-CoV-2 [68], A cytokine storm in the CNS may emerge from the simultaneous activation of several TLRs. TLRs contribute to PD by mediating the associated neuroinflammation and glial activation [69]. The interaction between α-synuclein and microglial TLR2 promotes the growth and spread of α-synuclein pathology [69].

### Endoplasmic reticulum stress and mitochondria

As SARS-CoV-2 simultaneously inhibited the expression of SELENOF, SELENOM, SELENOK, and SELENOS, the endoplasmic reticulum is an organelle that is badly damaged by the virus. Endoplasmic reticulum stress and the unfolded protein response are driven by coronavirus replication in infected cells [70–73]. Although endoplasmic reticulum-resident selenoproteins are known to have a role in preserving endoplasmic reticulum homeostasis, a connection between coronavirus infection and endoplasmic reticulum-resident selenoproteins has not yet been established [74]. Moreover, mitochondria have a role in the induction of the inflammatory response, including the production of mitochondrial reactive oxygen species(ROS) and the up-regulation of the expression of genes linked to glycolysis-related enzymes, which has also been extensively reported in the CNS in COVID-19 [75]. Dysregulation of the mitochondrial ACE2/MrgE/NO axis may have a significant effect on the neurodegenerative processes of dopaminergic neurons, where mitochondrial dysfunction and oxidative stress may have a substantial impact [76].

## Ferroptosis signature in SARS-CoV-2 infection and molecular mechanisms of ferroptosis

A case study of a COVID-19 patient has showed the presence of a ferroptosis signature in cardiac and renal tissues [77]. The finding was the first to document a ferroptosis signature in COVID-19, which was thought to be a risk factor for organ damage. Moreover, an in vitro investigation revealed that glutathione peroxidase 4 (GPX4), which was the brake of ferroptosis, was reduced by SARS-CoV-2 [78]. SARS-CoV-2 infects pacemaker cells easily, resulting in a noticeably increased rate of ferroptosis [79]. A growing body of research has revealed that ferroptosis plays significant pathogenetic roles in cancer, ischemia organ damage, and dementia since it was first used by Dixon et al. [80]. The precise mechanism underlying ferroptosis is still unknown, but it is known that altered iron metabolism, glutathione (GSH) depletion, GPX4 inactivation, and increased PUFA peroxidation by ROS play key roles in its onset and progression [80, 81]. In general, iron overload in cells, decreased GPX4 and xCT expression, activation of acylCoA synthetase long-chain family member-4 (ACSL4) and lysophosphatidylcholine acyltransferase-3, and an increase in lipid peroxidation are the four main mechanisms that induce ferroptosis [82–84].

## The potential role of ferroptosis underlies COVID-19-related PD

### Ferroptosis may exist in COVID-19-related PD

A potentially lethal aspect of SARS-CoV-2 infection is the involvement of neuropsychiatric symptoms, as was mentioned above. Ferroptosis has been identified as a key mechanism for the death of dopaminergic neurons in PD [85]. Severe behavioral impairment and neuronal death of mice given the ferroptosis inhibitor ferrostatin-1 24 h before 1-methyl- 4-phenyl-1, 2, 3, 6-tetrahydropyridine(MPTP) were greatly reversed [86]. Ferrostatin-1 also has a neuroprotective impact on SH-SY5Y cells injured by rotenone and 1-methyl-4-phenylpyridinium(MPP^+^) [87, 88], suggesting that ferroptosis could offer an alternative for treating PD. The enhanced ferrostatin-1 and liproxstatin-166 analogs, two members of the new generation of ferroptosis inhibitors, can be used to assess the involvement of ferroptosis in SARS-CoV-2 infection as well as to potentially treat COVID-19 [89, 90].

### Dysregulation of iron metabolism in COVID-19-related PD

Iron metabolism impairment, a significant contributor to PD [91, 92], has been extensively established in a significant fraction of COVID-19 patients in response to SARS-CoV-2 infection [93–95], which corresponds with the risk of severe and fatal COVID-19 illness. In our previous study, we elaborated the mechanism of dysregulation of iron metabolism and ferritinophagy in COVID-19 [96, 97]. In addition, ceruloplasmin levels in long-term COVID-19 patients exhibit a declining tendency when compared to those in COVID-19 patients and healthy controls [98]. PD is partly caused by the neurotoxicity of iron accumulation brought on by inadequate or reduced ferroxidase activity of ceruloplasmin [99, 100]. The accumulation of iron may cause a rise in the intracellular labile iron (II) pool and Fenton reaction, which results in the production of lipid ROS, and ferroptosis. Intracellular iron depletion would be potential treatment options for COVID-19. Deferoxamine and imatinib have been shown to prevent SARS-CoV-2 infection of pacemaker cells as well as SARS-CoV-2 infection-induced ferroptosis [79].

### GSH-GPX4 axis in COVID-19-related PD

**Fig. 1:**
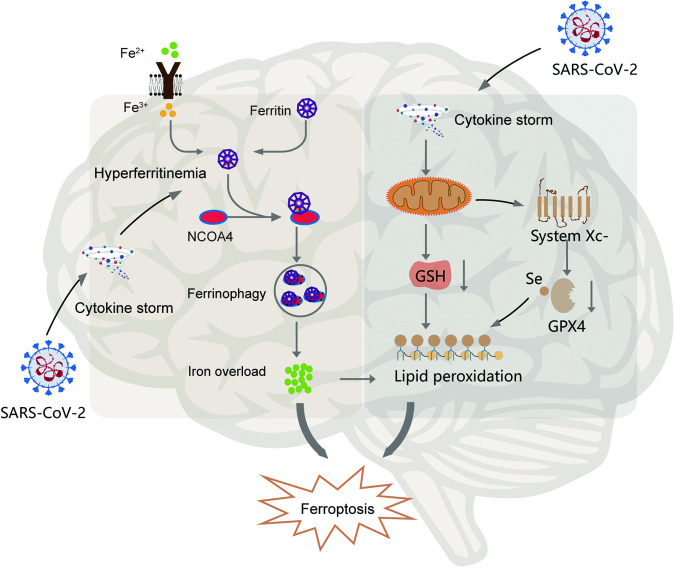
The involvement of ferroptosis in SARS-CoV-2 infection related to PD. SARS-CoV-2 infection may lead to ferroptosis for PD pathogenesis, probably contributing to the initiation of the disease through two major pathways: transporter-dependent pathway with iron imbalance and the intrinsic or enzyme regulated pathway.

Mitochondrial ROS production was increased by SARS-CoV-2 infection and its replication [101]. GPX4, located in the mitochondria, specifically guards against the ferroptotic cell death. GPX4 gene expression is suppressed by SARS-CoV-2, which promoted the occur of ferroptosis. A fundamental investigation that infected African green monkey kidney (Vero) cells with patient-derived SARS-CoV2 discovered that the mRNA levels of GPX4 were considerably downregulated, suggesting a connection between ferroptosis and SARS-CoV-2 [78]. Leukopenia in COVID-19 patients may be related to ferroptosis in leukocytes and suppressed GPX4 caused by SARS-CoV-2 [102].The lack of GPX4 induced the loss ability of GSH be peroxidized to minimize the lipid ROS produced by the Fenton reaction. Lipid peroxidation and ferroptosis would therefore follow from an accumulation of lipid ROS. Consequently, it’s probable that ferroptosis contributes to the PD symptoms of COVID-19 (Fig. 1).

## Discussion

### Challenges in establishing causal relationships between COVID-19 and PD

Many concerns still need to be addressed, despite the fact that numerous linked research about COVID-19 and PD are beginning to emerge. It is uncertain if distinct SARS-CoV-2 strains cause different neurological symptoms. To determine the connection between SARS-CoV-2 mutations and PD manifestations, further in-depth analyses are required. Moreover, it has been discovered that several forms of cell death, such as autophagy, apoptosis, and pyroptosis, are implicated in the pathogenic mechanism of both COVID-19 and PD [103, 104]. Since it is currently rather challenging to determine the cross-talk among different cell death pathways in COVID-19 related PD, none of the above mentioned mechanisms of cell death, other than ferroptosis, are explored in this review. Furthermore, the age and gender of the SARS-CoV-2 infection victim, which might be contributing variables to the development of PD, are not assessed. Moreover, it would be required to account for both environmental and genetic influences.

Both direct neuronal invasion and indirect effects of neuroinflammation may be involved in the neuropsychiatric symptoms of COVID-19. A deeper comprehension and functional characterize of COVID-19 related PD will be possible through the use of high throughput assessment and patient-derived organoids, which may provide a viable means of elucidating pathophysiologic hints and possible treatment approaches.

### The Potential clinical implications of ferroptosis in COVID-19 related PD

As a novel form of cell death, ferroptosis has great promise for study in COVID-19 associated with PD. A potential treatment approach might involve focusing on ferroptosis. So far, iron chelators and lipophilic antioxidants have been the principal approaches of suppressing ferroptosis [105]. Through the control of the Fenton reaction, iron chelators such as deferoxamine chelate iron and halt lipid peroxidation. It is important to note that iron alterations in the brain may be easily tested using quantitative susceptibility mapping [106]. Ferrostatin-1 and liproxstatin-1 are typical lipophilic antioxidants that scavenge lipid peroxides and inhibit ferroptosis. Studies have demonstrated that both iron chelators and lipophilic antioxidants could prevent the progression of PD [107]. Furthermore, deferoxamine lowers the amounts of IL-6, a major inflammatory cytokine generated during COVID-19 [108], suggesting that deferoxamine may be used as a medication to treat COVID-19-induced PD. Nevertheless, no study has yet evaluated the effectiveness of lipophilic antioxidants in the COVID-19 therapy process. Moreover, future research should look into the potential benefits of combining anti-inflammatory cytokines with ferroptosis interference to improve the resilience of COVID-19-related PD patients, considering that inflammatory cytokine storms are believed to be key contributors to COVID-19.

To the best of our knowledge, neither clinical trials assessing ferroptosis inhibitors in COVID-19 related PD nor any indication of the ferroptosis signature in the brain tissues of COVID-19 patients exist. Though we have made great progress in understanding the pathogenic role of ferroptosis in PD, the precise role that ferroptosis plays in the brain damaged by SARS-CoV-2 and how it initiates the inflammation that ultimately causes brain damage is unclear. In addition, it is hard to describe ferroptosis is a side effect of SARS-CoV-2 infection or if it’s a way for the virus to replicate and become more dangerous during COVID-19. Despite we have elucidated the connection between three primary ferroptosis pathways and COVID-19, which path is more important in COVID-19-related brain damage? It is necessary to address these issues in order to make a complete and convincing argument for the therapeutic use of ferroptosis inhibitors.

## Conclusion remark

**Fig. 2:**
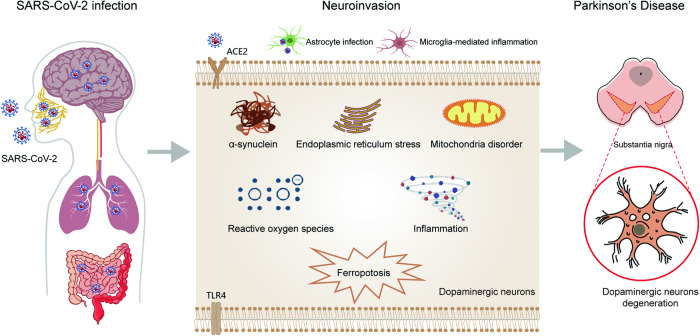
Mechanisms of pathogen invasion and its potential contribution to the PD. SARS-CoV-2 might infiltrate the CNS directly through the olfactory, respiratory tract, gastrointestinal tract, blood-brain barrier. The infection could prompt cytotoxic aggregation of α-synuclein, endoplasmic reticulum stress, mitochondria damage, neuroinflammation, and ferroptosis, which induce dopaminergic neurons degeneration.

From the beginning of the epidemic, scientists have been working feverishly to discover a new COVID-19 vaccine or possible treatment. This review generally overviews the relationship between COVID-19 and PD (Fig. 2). The course of the COVID-19 and PD exhibit similarities in some biochemical processes, including oxidative stress, inflammation, and protein aggregation [53]. Since that COVID-19 exhibits unusual symptoms including GSH depletion, GPX4 inactivation, abnormal iron metabolism, and elevation of PUFA peroxidation by reactive oxygen species, it is possible that SARS-CoV-2 might cause ferroptosis in the dopaminergic neurons, which would then contribute to PD. Therefore, this review presents evidence that ferroptosis is intimately linked to and holds considerable promise for research on COVID-19-related PD, which provide a promising research direction. We speculate that ferroptosis contributes to SARS-CoV-2 infection-related PD in light of the possible link between ferroptosis and neurological abnormalities in COVID-19 patients. Although there is a lack of evidence on effective treatment strategies for COVID-19-related PD, one potentially effective tactic would be to target ferroptosis. However, it is yet unknown how ferroptosis functions in SARS-CoV-2 infected dopaminergic neurons or whether it represents a promising new therapeutic target for COVID-19-related PD therapy. Further researches are aggressively explored to confirm that ferroptosis occurs in COVID-19, clarify its precise mechanism, and determine if it is linked to brain damage associated with COVID-19.

## Methods

Relevant articles were reviewed in databases of PubMed and Web of Science. To identify eligible studies, we searched and exploded the following key terms and combinations: (“COVID-19” or “SARS-CoV-2”) and (“Parkinson’s disease” or “PD”) and/or (“Ferroptosis”). We also identified related publications written in Chinese from China National Knowledge Infrastructure (CNKI) database and Wanfang using the above combinations terms in Chinese. The search was conducted in Oct 1, 2023.

### Inclusion and exclusion criteria

The inclusion criteria were as follows: (1) full-text articles; (2) providing sufficient data about the links between COVID-19 neurotropism and PD; (3) iron underlies COVID-19-related PD; (4) GSH-GPX4 Axis in COVID-19-related PD.

Studies were excluded for the following reasons: (1) irrelevant papers of COVID-19-related psychiatric symptoms; (2) papers about COVID-19 vaccine; (3) nursing care/management of COVID-19 patients; (4) social and psychological impact of COVID-19; (5) sleep disturbances of COVID-19; (6) lockdown effects/impact of home confinement;

### Search selection

Initial screening of total retrieved articles was made by title and abstract. Authors (FJ) then performed a full-text evaluation of relevant articles and of articles where the abstract did not provide sufficient information. The search was conducted independently by the authors (JH) and compared against each other to identify/discuss discrepancies. Articles which did not meet inclusion criteria were excluded.

### Supplementary information

Checklist
